# Fetal Echocardiographic Dimension Indices: Important Predictors of Postnatal Coarctation

**DOI:** 10.1007/s00246-020-02509-6

**Published:** 2020-12-23

**Authors:** Katrin Fricke, Petru Liuba, Constance G. Weismann

**Affiliations:** Lund University, Skane University Hospital, Department of Clinical Sciences Lund, Pediatric Cardiology, Lund, Sweden

**Keywords:** Fetus, Coarctation, Fetal echocardiography, Carotid-subclavian artery index, Prenatal diagnosis

## Abstract

The aim of the study is to identify reliable quantitative fetal echocardiographic predictors for postnatal development of coarctation (CoA). In this retrospective study, we included 65 fetuses with a prenatally suspected, isolated CoA, born 2010–2018. Dimensions of the cardiac structures, aortic, and ductal arches expressed as ratios and Z-scores were analyzed in relation to outcome. Fetuses that developed CoA postnatally (34%) exhibited significantly smaller Z-scores of left cardiac structures from the mitral valve to the aortic isthmus. The most sensitive and specific predictors were a carotid-subclavian artery index (CSAI) of < 0.78 (92.3% sensitivity, 96.8% specificity) or a product of isthmus-to-duct ratio in the three-vessel trachea view (3VT) and the mitral-to-tricuspid valve ratio (I/D_3VT_xMV/TV) of < 0.37 (100% sensitivity, 94.6% specificity). When comparing different Z-score datasets, we observed large and highly significant differences. Postnatal CoA can be predicted with high accuracy during fetal life using CSAI or I/D_3VT_xMV/TV. The latter may be particularly useful if adequate sagittal aortic arch images cannot be obtained. As significant and clinically unacceptable differences in Z-scores were observed for the same measurements, this calls for a large multi-center collaboration to generate reliable fetal echocardiographic Z-scores.

## Introduction

Coarctation of the aorta (CoA) is one of the most common congenital heart defects (CHDs) accounting for 8% of all CHDs [1]. It is defined as a circumscribed narrowing of the aortic isthmus, often combined with a tubular hypoplasia of the aortic arch. CoA may occur in isolation or in association with other CHDs, most commonly ventricular septal defects (VSD). If not detected prenatally or shortly post partum, critical narrowing of the aortic isthmus may develop as the arterial duct closes. This leads to underperfusion of the lower body and subsequently to multiorgan failure, shock, and even death. If CoA is diagnosed prior to clinical decompensation, however, the prognosis following surgical repair is generally good [2].

Thus, a timely, preferably prenatal diagnosis is essential in order to facilitate delivery planning and avoid unnecessary morbidity and mortality [3].

Unfortunately, the prenatal diagnosis of CoA remains challenging in spite of technical advances. Fetal CoA is often suspected in case of a disproportion of the ventricles and great vessels with smaller left heart structures. Overall, fewer than 50% of fetuses with a ventricular disproportion develop CoA postnatally, leading to high false-positive detection rates associated with unnecessary parental anxiety and use of healthcare-related resources [4–7]. On the flipside, prenatal detection rates for CoA are only approximately 20–35% and thereby among the lowest of all critical CHDs [8–10]. This low prenatal detection rate is related to the fact that the actual narrowing of the isthmus typically does not develop until the arterial duct closes postnatally. Even pulse oximeter newborn screening has only marginally improved the early detection rate for CoA [11, 12].

It was the primary aim of this study to identify reliable quantitative fetal echocardiographic predictors of postnatal CoA, using both dimension ratios and Z-scores of cardiac structures and the secondary aim to compare Z-scores calculated from various normative datasets.

We hypothesized that development of CoA postnatally can be predicted with high diagnostic accuracy.

## Methods

This retrospective study was conducted at Skane`s University Hospital at Lund University, one of two tertiary referral centers for pediatric cardiac surgery in Sweden. The institutional fetal cardiology database and “The Swedish Registry of Congenital Heart Defects” (SWEDCON) were searched for infants born 2010–2018 with a prenatal suspicion of CoA. Fetuses with prenatally suspected CoA with or without borderline hypoplasia of the left heart structures were included. Exclusion criteria were prenatally suspected hypoplastic left heart syndrome, complex CHD other than associated aortic arch hypoplasia, VSD, mild aortic (AS) or mitral valve stenosis (MS) or persistent left superior vena cava (LSVC), and insufficient technical quality including suboptimal imaging of the sagittal aortic arch. This study was approved by the Regional Ethical Review Board according to the Helsinki declaration.

Demographic pre- and postnatal variables were obtained from the medical record and operative reports. If multiple echocardiograms were available, the study with the most optimal imaging technique was used for analysis.

Fetal echocardiograms were analyzed using SyngoDynamics (Siemens, Germany). All measurements were conducted by an experienced fetal echocardiographer (K.F.).

### Fetal Echocardiographic Measurements

Quantitative measurements of fetal echocardiograms included dimensions of the left and right cardiac structures and function as well as standard and non-standard measurements of the aortic arch and arterial duct. According to published guidelines, fetal Z-scores corresponding to gestational age were computed [13–16].

The following ratios between left and right cardiac structures were determined: left-to-right ventricular (LV/RV) width and length ratios and mitral-to-tricuspid valve (MV/TV) dimension ratio examined in the four-chamber view; aortic-to-pulmonary valve (AoV/PV) ratio; ascending-to-descending aorta (Ao asc/DAo) ratios measured in the outflow tract and sagittal views; and isthmus aortae-to-arterial duct (I/D) diameter ratios examined in the three-vessel trachea (3VT) and sagittal views. In addition, we calculated the carotid-subclavian artery index (CSAI), defined as the ratio of the aortic arch diameter at the left subclavian artery, to the distance between the left carotid artery and the left subclavian artery [17].

Moreover, we examined anatomy and shunt direction through the interatrial communication and aortic arch. Morphological and functional features of the ventricles and aortic arch as well as the presence of associated minor CHDs were noted.

In addition, we validated the most important study results prospectively in 16 fetuses with prenatally suspected CoA born in 2019.

### Post Partum Approach if CoA is Suspected in Fetal Life

Newborns with prenatally suspected CoA are delivered vaginally in our tertiary center with unimpeded access to cardiac surgery if needed. In the majority of cases, infants are monitored post partum for the development of coarctation without starting prostaglandins. When developing CoA is confirmed clinically and echocardiographically, prostaglandin infusion is started and maintained until surgical repair. In isolated cases, when there is marked hypoplasia of the isthmus and/or aortic arch, we refrain from a trial-off prostaglandin.

### Statistics

Data are presented as median (inter-quartile range) or mean (standard deviation). Group-wise comparisons of fetuses with and without postnatal CoA were performed using the Chi^2^- or Fisher´s Exact test for categorical variables and Mann–Whitney-U-test for continuous variables. T-test for related samples was used when comparing different Z-score datasets. Logistic regression analyses were performed using postnatal development of CoA as the dependent variable and quantitative echocardiographic measures as independent variables. Odds ratios (95% confidence interval) are provided. Receiver operating characteristic (ROC) curves and area under the curve (AUC) were calculated for variables significantly associated with the postnatal development of CoA. Cut-off points with corresponding sensitivity and specificity are provided. Study data were collected and managed using REDCap electronic data capture tools hosted at Lund University [18, 19]. Statistical analyses were performed using Statistical Package for Social Sciences, version 25 (IBM SPSS, Chicago, IL).

## Results

Seventy-seven fetuses with prenatally suspected CoA born between 2010 and 2018 were identified. There was no intrauterine death. Twelve cases were excluded due to technically inadequate documented sagittal clips of the aortic arch. Of the remaining 65 fetuses, 22 (34%) developed CoA postnatally, whereof 4 underwent univentricular palliation due to associated mitral and/or aortic valve pathologies. All patients with postnatally confirmed CoA or univentricular circulation were operated in the neonatal period: 4 with end-to-end anastomosis, 11 with end-to-side anastomosis, 3 with arch reconstruction, and 4 with stage I Norwood palliation.

The most common indication for the initial fetal echocardiogram was an abnormal second or third trimester obstetrical screening ultrasound (*n* = 60), usually due to a disproportion of the ventricles or great vessels. Other indications included an abnormal first trimester screening (*n* = 2), maternal diabetes (*n* = 5), fetal arrhythmias (*n* = 2), or family history of CHD (*n* = 2). Forty-five fetuses (69.2%) had a high suspicion for CoA in the final echocardiogram prior to delivery according to the evaluating fetal cardiologist.

The mean gestational age at the time of the fetal echocardiogram was 34.1 weeks (range 26.3–39.3). Forty-four fetuses (68%) exhibited a moderately underdeveloped left ventricle (borderline left ventricle), defined as Z-scores for width of −2 to −4 and with antegrade flow across the mitral and aortic valve. Associated cardiac anomalies included a hypoplastic aortic arch based on qualitative assessment (*n* = 22), LSVC (*n* = 7), mild MS (*n* = 1) or AS (*n* = 2), and VSD (*n* = 10). Three fetuses had associated extracardiac anomalies, and one had a genetic anomaly (Down`s syndrome).

### Qualitative Fetal Echocardiographic Assessment

A bidirectional or retrograde flow in the aortic arch (*p* < 0.001) or bidirectional or left–right shunt across the interatrial communication (*p* = 0.002) was linked to postnatal CoA (Table 1). Furthermore, postnatal CoA was significantly associated with qualitative assessment of a borderline hypoplastic left ventricle, hypoplastic aortic arch, posterior shelf, and VSDs detected on the fetal echocardiogram (Table 1).

**Table 1:** Categorical parameters in fetuses with and without postnatal CoA

Categorical parameters	Postnatal CoA n/N (%)	No postnatal CoA n/N (%)	*P* value
Interatrial shunt direction			
Right-left	2/17 (11.8)	22/34 (64.7)	0.002
Left-right	1/17 (5.9)	0/34 (0)	
Bidirectional	13/17 (76.5)	12/34 (35.3)	
No shunt	1/17 (5.9)	0/34 (0)	
Flow direction aortic arch			
Antegrade	6/20 (30)	32/43 (74.4)	< 0.001
Bidirectional	9/20 (45)	11/43 (25.6)	
Retrograde	5/20 (25)	0/43 (0)	
Borderline left ventricle	21/22 (95.5)	23/43 (53.5)	0.001
Hypoplastic aortic arch	20/22 (90.9)	2/43 (4.7)	< 0.001
Posterior Shelf	14/15 (93)	12/39 (30.8)	< 0.001
Left superior vena cava	4/22 (18.2)	3/43 (7)	0.17
Ventricular septal defect	8/22 (36.4)	2/43 (4.7)	0.002

*CoA* coarctation, *n* number of patients for given variable, *N* total number of patients

*N* = 65, for interatrial shunt direction = 51; for flow direction aortic = 63; for posterior shelf = 54

### Quantitative Fetal Echocardiographic Measurements

Fetuses with a postnatally confirmed CoA exhibited significantly smaller left cardiac structures from the mitral valve to the aortic isthmus when adjusted for gestational age (Table 2). Notably, both groups had median Z-scores below average, i.e., Z-score < 0. Tricuspid and pulmonary valve Z-scores as well as right ventricular length and arterial duct Z-scores were not significantly different between the groups. Of the right cardiac structures, only right ventricular width was significantly larger in fetuses with postnatally confirmed CoA. Lastly, all ratios between left and right cardiac structures, except for LV/RV length ratio, were significantly lower in fetuses with postnatally confirmed CoA (Table 2).

**Table 2:** Z-scores and ratios of continuous fetal echocardiographic parameters

Continuous parameters		Postnatal CoA median (IQR)	No postnatal CoA median (IQR)	*P* value
Left heart structures (Z-score sets)				
Mital valve annulus	Krishnan et al. [14]	–3.35 (–4.68 to –2.20)	–1.05 (–1.80 to –0.17)	< 0.001
	Schneider et al. [16]	–4.00 (–5.43 to –3.07)	–1.85 (–2.63 to –1.00)	< 0.001
Left ventricular length	Krishnan et al. [14]	–3.95 (–4.78 to –2.69)	–3.00 (–3.60 to –2.50)	0.009
	Schneider et al. [16]	–1.70 (–2.30 to –0.65)	–0.90 (–1.20 to –0.40)	0.002
Left ventricular width	Gabbay–Benziv et al. [13]	–2.25 (–3.40 to –1.87)	–0.80 (–1.45 to –0.45)	< 0.001
	Schneider et al. [16]	–1.70 (–2.70 to –1.20)	–0.60 (–1.10 to– 0.00)	< 0.001
Aortic valve annulus	Krishnan et al. [14]	–2.55 (–2.95 to –1.00)	–0.06 (–0.70 to– 0.60)	< 0.001
	Schneider et al. [16]	–2.75 (–3.13 to –1.53)	–0.70 (–1.20 to –0.10)	< 0.001
Ascending aorta	Krishnan et al. [14]	–3.10 (–4.45 to –1.95)	–1.50 (–1.90 to –0.75)	< 0.001
	Schneider et al. [16]	–3.60 (–4.45 to –2.15)	–1.40 (–1.90 to–0.90)	< 0.001
Isthmus aortae (sag.)	Krishnan et al. [14]	–4.70 (–6.4 to –3.95)	–2.60 (–3.70 to –1.40)	< 0.001
	Pasquini et al. [15]	–2.40 (–4.15 to –1.85)	–1.00 (–1.80 to –0.50)	< 0.001
Isthmus aortae (3VT)	Pasquini et al. [15]	–3.00 (–4.05 to –2.70)	–1.70 (–2.40 to –0.68)	< 0.001
Descending aorta	Krishnan et al. [14]	–1.05 (–1.47 to –0.63)	–0.90 (–1.40 to –0.52)	0.26
	Schneider et al. [16]	–0.35 (–0.78–0.28)	–0.03 (–0.60–0.35)	0.22
Right heart structures (Z-score sets)				
Tricuspid valve annulus	Krishnan et al. [14]	0.30 (–0.70–0.85)	0.50 (–0.12–0.90)	0.34
	Schneider et al. [16]	–0.40 (–1.13–0.33)	–0.10 (–0.80–0.43)	0.33
Right ventricular length	Krishnan et al. [14]	–2.40 (–3.35 to –1.75)	–1.90 (–2.90 to –1.20)	0.11
	Schneider et al. [16]	–0.70 (–1.33 to –0.08)	–0.20 (–0.90–0.25)	0.06
Right ventricular width	Gabbay–Benziv et al. [13]	0.95 (0.00–2.12)	0.08 (–0.35–1.00)	0.02
	Schneider et al. [16]	0.99 (0.15–1.85)	0.19 (–0.23–1.00)	0.02
Pulmonary valve annulus	Krishnan et al. [14]	1.40 (0.50–2.60)	1.70 (1.00–2.55)	0.79
	Schneider et al. [16]	0.60 (–0.05–1.90)	1.00 (0.25–1.90)	0.74
Arterial duct (sag.)	Krishnan et al. [14]	–1.30 (–2.24– 0.10)	–2.30 (–2.70 to –1.50)	0.05
	Schneider et al. [16]	1.15 (1.00– 1.92)	1.00 (0.50–1.50)	0.07
Arterial duct (3VT)	Pasquini et al. [15]	1.00 (0.07–1.58)	0.25 (–0.60–1.10)	0.12
Ratios				
Left/right ventricular length		0.96 (0.85–1.04)	1.00 (0.92–1.11)	0.13
Left/right ventricular width		0.60 (0.52–0.72)	0.82 (0.73–0.88)	< 0.001
Mitral/tricuspid valve annulus		0.55 (0.47–0.52)	0.73 (0.67–0.82)	< 0.001
Aortic/pulmonary valve annulus		0.54 (0.46–0.59)	0.66 (0.60–0.71)	< 0.001
Carotid- subclavian artery index		0.51 (0.43–0.58)	1.35 (1.05–1.88)	< 0.001
Ascending/descending aorta		0.84 (0.61–0.94)	0.96 (0.92–1.01)	< 0.001
Isthmus/arterial duct (sag.)		0.43 (0.38–0.54)	0.66 (0.59–0.73)	< 0.001
Isthmus/arterial duct (3VT)		0.48 (0.43–0.53)	0.67 (0.61–0.75)	< 0.001

*CoA:* coarctation, *3VT view:* three-vessel trachea view; *sag.* sagittal view

Measurements of the arterial duct in sagittal (*n* = 55) or 3VT view (*n* = 57). All other cardiac structures could be measured in 62 or more cases

**Fig. 1:**
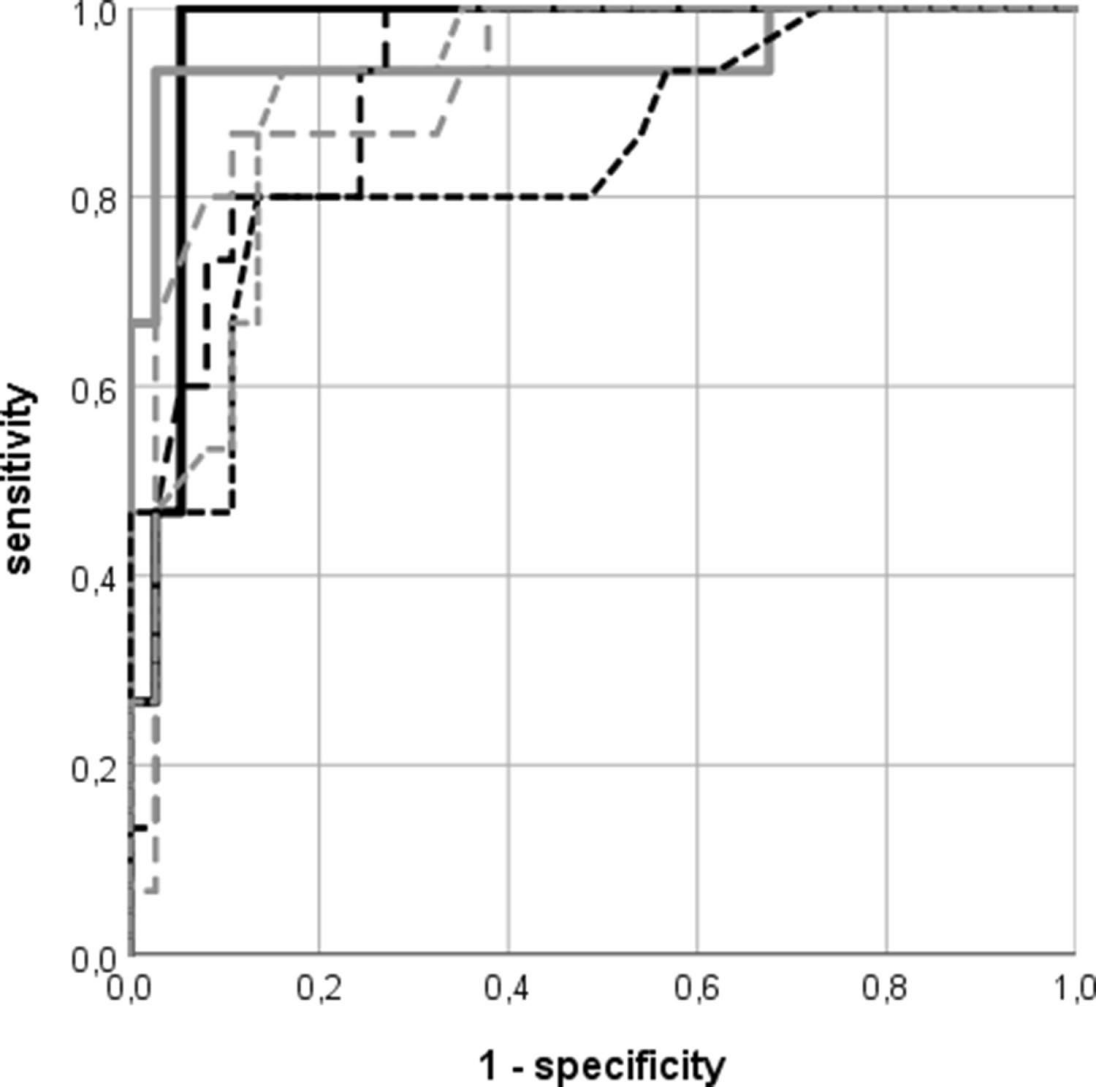
Receiver operation characteristic (ROC) curve for selected fetal echocardiographic variables using postnatal development of aortic coarctation as the outcome: black continuous line: product of isthmus/arterial duct in the three-vessel trachea view and mitral/tricuspid valve in the four-chamber view (I/D_3VT_xMV/TV); gray continuous line: carotid-subclavian artery index; black dashed line: isthmus-ductus ratio in the three-vessel trachea view; gray dashed line: mitral/tricuspid valve annulus index; black dotted line: isthmus Z-score in the three-vessel trachea view; gray dotted line: mitral valve annulus Z-score (Krishnan et al. [14])

For parameters that were significantly different in the group-wise comparison, logistic regression analyses and receiver operation characteristics were carried out (Table 3). Due to the high number of parameters significantly associated with postnatal CoA, we present data on specificity and sensitivity as well as suggested cut-off points only for variables with an area under the curve of at least 0.90 (Table 4). The parameter with the highest sensitivity and specificity was the CSAI under 0.78 with 92.3% and 96.8%, respectively. Alternatively, combining the I/D ratio in the 3VT view with the MV/TV ratio in the four-chamber view allows for comparable, if not superior sensitivities and specificities (Table 4, Fig. 1). Both ratios were measureable in the majority of fetuses (*n* = 53 of 65, 81.5%).

**Table 3:** Odds ratios and area under the curve for significant Z-scores and ratios

Continuous parameters		Postnatal CoA			
		OR (95% CI)	*P* (OR)	AUC (95% CI)	*P* (AUC)
Left heart structures (Z-score sets)					
Mitral valve annulus	Krishnan et al. [14]	0.16 (0.06–0.44)	< 0.001	0.94 (0.89–0.99)	< 0.001
	Schneider et al. [16]	0.39 (0.23–0.65)	< 0.001	0.88 (0.77–0.99)	< 0.001
Left ventricular length	Krishnan et al. [14]	0.49 (0.30–0.82)	0.006	0.68 (0.53–0.83)	0.019
	Schneider et al. [16]	0.36 (0.18–0.70)	0.003	0.72 (0.58–0.87)	0.004
Left ventricular width	Gabbay-Benziv et al. [13]	0.26 (0.13–0.51)	< 0.001	0.82 (0.67–0.97)	0.001
	Schneider et al. [16]	0.27 (0.14–0.55)	< 0.001	0.78 (0.62–0.94)	0.004
Aortic valve annulus	Krishnan et al. [14]	0.28 (0.14–0.53)	< 0.001	0.79 (0.60–0.97)	0.003
	Schneider et al. [16]	0.22 (0.10–0.47)	< 0.001	0.78 (0.59–0.97)	0.004
Ascending aorta	Krishnan et al. [14]	0.28 (0.14–0.55)	< 0.001	0.82 (0.69–0.95)	< 0.001
	Schneider et al. [16]	0.21 (0.09–0.47)	< 0.001	0.85 (0.72–0.97)	< 0.001
Isthmus aortae (sag.)	Krishnan et al. [14]	0.45 (0.29–0.68)	< 0.001	0.81 (0.67–0.95)	0.001
	Pasquini et al. [15]	0.25 (0.12–0.52)	< 0.001	0.85 (0.73–0.97)	< 0.001
Isthmus aortae (3VT)	Pasquini et al. [15]	0.21 (0.09–0.52)	0.001	0.80 (0.66–0.96)	0.001
Right heart structures Z-score sets					
Right ventricular width	Gabbay-Benziv et al. [13]	1.72 (1.07–2.75)	0.024	0.68 (0.53–0.82)	0.019
	Schneider et al. [16]	1.93 (1.09–3.40)	0.025	0.68 (0.53–0.82)	0.016
Ratios					
Left/right ventricular width		1 ×10^−6^(1.2 × 10^–9^−0.001)	< 0.001	0.89 (0.79–0.99)	< 0.001
Mitral/tricuspid valve annulus		1.08 ×10^-8^(9.4 × 10^−13^-1.2 ×10^−4^)	< 0.001	0.90 (0.8–0.99)	< 0.001
Aortic/pulmonary valve annulus		3.1×10^−10^(2.6×10^−15^–(2.6×10^−15^3.8×10^−5^)	< 0.001	0.79 (0.66–0.93)	0.002
Ascending/descending aorta		1.3×10^−^(2 × 10^−8^−0.009)	0.001	0.69 (0.52–0.86)	0.05
Carotid-subclavian artery index		0.001 (3.2 ×10^−5^–0.034)	< 0.001	0.94 (0.85–1)	< 0.001
Isthmus/arterial duct (sag.)		4×10^−7^(2×10^−10^−0.001)	< 0.001	0.90 (0.81–0.99)	< 0.001
Isthmus/arterial duct (3VT)		4×10^−9^(1.3×10^−13^–1.2×10^−4^)	< 0.001	0.93 (0.85–1)	< 0.001

*OR (95% CI)*: Odds ratio with 95% confidence interval, *AUC (95% CI)*: area under the curve with 95% confidence interval, *sag.* sagittal view, *3VT:* three-vessel trachea view

**Table 4:** Cut-off points, sensitivity, and specificity for highly significant continuous parameters

Continuous parameters	Cut-off points	Sensitivity (%)	Specificity (%)
Left heart structures (Z-score sets)			
Mitral valve annulus: Krishnan et al. [14]	–2.03	90.5	88.1
Ratios			
Mitral/tricuspid valve annulus	0.61	84.6	87.1
Carotid-subclavian artery index	0.78	92.3	96.8
Isthmus/arterial duct (sag.)	0.59	92.3	77.4
Isthmus/arterial duct (3VT)	0.57	84.6	90
I/D_3VT_xMV/TV	0.37	100	94.6

*I/D*_*3VT*_*xMV/TV*: product of isthmus/arterial duct in the three-vessel trachea view and mitral/tricuspid valve in the four-chamber view

In a logistic regression model including both the I/D_3VT_xMV/TV and the CSAI, no interaction was noted (*p* = 0.6).

### Prospective Validation

Furthermore, we validated the CSAI and I/D_3VT_xMV/TV indices prospectively in 16 fetuses with prenatal suspicion of CoA born in 2019, four of which developed CoA postnatally. A CSAI < 0.78 detected postnatal development of CoA with a sensitivity of 100% and a specificity of 91.7%. The I/D_3VT_xMV/TV < 0.37 exhibited both a 100% sensitivity and specificity.

### Variability Between Z-Score Datasets

Lastly, we compared the reliability of different Z-score datasets on 12 parameters from the current study (Table 5). Significant differences between Z-score datasets were detected for right and left cardiac structures with mean differences up to 2.91 Z-scores for the same measurement (Table 5). Intraclass correlations coefficients comparing different Z-score datasets though were consistently above 0.93 (*p* < 0.001) except for arterial duct dimensions from the sagittal view (0.89, *p* < 0.001).

**Table 5:** Differences of Z-score sets for cardiac structures

Z-score sets	Mean difference (SD)	*P* value
Left heart structures		
Mitral valve annulus (Krishnan-Schneider)	0.68 (0.49–0.87)	< 0.001
Left ventricular length (Krishnan-Schneider)	–2.20 (–2.29 to –2.11)	< 0.001
Left ventricular width (Gabbay-Benziv-Schneider)	–0.42 (–0.49 to –0.36)	< 0.001
Aortic valve annulus (Krishnan-Schneider)	0.53 (0.46–0.59)	< 0.001
Ascending aorta (Krishnan-Schneider)	0.05 (–0.02–0.13)	0.17
Isthmus aortae (sag.) (Krishnan-Pasquini)	–1.59 (–1.83 to –1.36)	< 0.001
Descending aorta (Krishnan-Schneider)	–0.78 (–0.82 to –0.74)	< 0.001
Right heart structures		
Tricuspid valve annulus (Krishnan-Schneider)	0.57 (0.51–0.62)	< 0.001
Right ventricular length (Krishnan-Schneider)	–1.73 (–1.83 to –1.63)	< 0.001
Right ventricular width (Gabbay-Benziv-Schneider)	–0.003 (–0.06- 0.06)	0.92
Pulmonary valve annulus (Krishnan-Schneider)	0.68 (0.65–0.72)	< 0.001
Ductus arteriosus (sag.) (Krishnan-Schneider)	–2.91 (–3.10 to –2.71)	< 0.001

*SD:* standard deviation

## Discussion

Fetal prediction of postnatal CoA continues to be a challenge. We have demonstrated in this retrospective study of fetuses with suspected CoA that postnatal CoA can be predicted with a high degree of accuracy using indices with the fetus as the internal control. Specifically, those were the CSAI and a combined score of I/D in the 3VT view and MV/TV ratio in the four-chamber view. The use of Z-scores—using normal fetuses as controls—was limited by its lower predictive value, and potential differences related to “normal” cohorts and/or observers.

### Z-Scores

Our study showed that fetuses with postnatally confirmed CoA exhibited significantly smaller left cardiac structures and diameters of the isthmic region normalized for gestational age. The best Z-scores for prediction of postnatal CoA were those of the MV-annulus, ascending aorta, and isthmus aortae in the sagittal or 3VT views, which have been described in other studies [6, 20–24]. Most studies to date have used Z-scores from Pasquini et al. for isthmic or ductal diameters and Schneider et al. for intracardiac structures or the ascending aorta, often with a pre-defined cut-off point of < −2 [15, 16]. The cut-off points calculated in our study are deviated for most anatomic structures from −2. Furthermore, we only included fetuses with prenatally suspected CoA in our study, which likely explains why left cardiac structures generally had Z-scores < 0 (−0.6 to −3) even in fetuses who did not develop postnatal CoA.

### Ratios

Various ratios of the left-to-right cardiac structures have been reported as sensitive indicators for postnatal development of CoA [6, 25–27]. In our study, the best predictors for postnatal CoA were the aortic-arch-related ratios (CSAI and I/D ratios) and MV/TV ratio.

Our study showed that the application of the sagittal arch view for depiction of the aortic and ductal arch is important for risk stratification of fetuses with prenatal suspicion of CoA. The CSAI had the highest sensitivity and specificity for detection of postnatal CoA (Table 4). The CSAI has previously been described to predict the development of CoA in newborns [17]. Further, two fetal studies have suggested that CSAI is a reliable predictor for postnatal CoA [23, 28]. However, both studies have smaller sample sizes and did not specify cut-off points with corresponding sensitivity and specificity, making it difficult to guide risk stratification for postnatal development of CoA in those fetuses. The current study is the first to demonstrate superiority of CSAI over Z-scores and other ratios in isolation for prediction of postnatal CoA.

The I/D ratio, which has previously been associated with the development of postnatal CoA, had a lower sensitivity and specificity compared to CSAI [5, 6, 24, 29].

CSAI can only be demonstrated from a sagittal view, which can be difficult to obtain due to fetal position or shadowing of the echogenic spine late in gestation. We therefore sought to combine measurements that do not require a sagittal aortic arch view to predict postnatal CoA. In fact, combining I/D ratio from the 3VT view with the MV/TV ratio in the four-chamber view, is an excellent alternative for prenatal prediction of CoA. The I/D_3VT_xMV/TV might be slightly superior to the CSAI, a finding we even could validate prospectively in fetuses with suspected CoA born in 2019. Its high accuracy might derive from combining both ventricular and isthmic-to ductal discrepancies in only one index. Because the I/D_3VT_xMV/TV index is technically easier obtained in pregnant women with suboptimal acoustic windows, this might reduce the frequency of clinical consultations and in its turn healthcare costs and anxiety of prospective parents.

Overall, ratios predicted postnatal CoA more reliably than Z-scores. We believe that using the individual fetuses cardiac structure as an internal control has the advantage of eliminating a) potential confounders related to comparison with “normal” cohorts (cardiac Z-scores are based on gestational age, not actual somatic size) and/or b) observer related subtle differences in measurement technique (ratios have the individual observers measurements as an internal control; Z-scores are based on measurements by other observers).

Qualitative variables have previously been associated with CoA and were not the focus of this study. Borderline left ventricular hypoplasia, a hypoplastic aortic arch, or posterior shelf are known predictors for postnatal CoA [6, 20, 29, 30]. We observed similar findings in our study. In the clinical setting, however, subjective criteria may generally be subject to higher inter-observer variability than quantitative data.

In addition, the association between the flow direction across the atrial septum or in the aortic arch and postnatal CoA has been reported [22, 23]. However, in our study, they had a relatively low specificity of 65–75%, leading in some cases to the misjudgment by the fetal cardiologist that there might exist high risk for postnatal CoA or even univentricular outcome, if the flow direction was bidirectional or left–right at the atrial level or bidirectional in the aortic arch.

A prenatally detected VSD in association with a suspicion for CoA was associated with postnatal CoA with a high specificity (90.7%) but a low sensitivity (36.4%). While the presence of a VSD in the setting of a suspected CoA is a red flag, its absence should not provide false reassurance [30].

### Differences Between Z-Scores

Perhaps most importantly clinically, we demonstrate significant differences between Z-scores based on Krishnan et al. [14] vs. Schneider et al. [16] or Pasquini et al. [15] and Gabbay-Benziv et al. [13] (Tables 2, 5) [13–16]. This was particularly evident for right and left ventricular length as well as sagittal isthmus aortae and arterial duct dimension Z-scores. Overall, the number of fetuses in each Z-score dataset is rather limited. The large volume of up to 414 fetuses has been published by Krishnan et al. [14]. We suggest that larger multi-center fetal echocardiographic data need to be analyzed in order to generate more reliable Z-score datasets. Meanwhile, for the individual patient, we recommend comparing the results of different Z-score datasets and interpreting them with caution in the context of the qualitative impression by the experienced fetal echocardiographer.

### Limitations

This study has several limitations including that it is a retrospective single-center study of small to moderate size with some missing data points (Table 1). The single fetal echocardiographer performing the measurements (K.F.) had clinical contact with some of the patients, so that bias could not be excluded. However, we were able to prospectively validate the findings using a completely blinded approach.

Moreover, we included fetal echocardiograms at later gestational age than the usual screening echocardiogram (18–24 weeks of gestation). Prospective multicenter validation of our findings, even in younger fetuses, is necessary prior to implementing our findings into clinical practice. In the future, we may test those indices even in fetuses with suspicion of CoA in more complex cases of CHD.

### Conclusion

It may be possible to predict postnatal CoA in third trimester fetuses with a prenatal suspicion with high accuracy. In the study presented herein, ratios were more reliable indicators than Z-scores, whereof the CSAI or alternatively a combination of the isthmus aortae-to-arterial duct ratio in the 3VT view with the mitral-to-tricuspid valve ratio was most predictive. The I/D_3VT_xMV/TV index is a new index that might be superior to the CSAI when sagittal arch views are difficult to obtain. Its high accuracy might derive from combining both ventricular and isthmic-ductal discrepancies in one index. In addition, using the available fetal Z-score datasets, significant and clinically unacceptable differences in Z-scores were observed for the same measurements. This calls for a large multi-center collaboration to generate reliable fetal echocardiographic Z-scores.
